# Golden eagle optimized fractional-order PI controller design for a PFC SEPIC converter in EV charging

**DOI:** 10.1038/s41598-024-69653-4

**Published:** 2024-09-09

**Authors:** S. Vijayakumar, N. Sudhakar

**Affiliations:** School of Electrical Engineering, Vellore Institute of Technology, Vellore, 632014 India

**Keywords:** FOPI, Power factor correction, SEPIC converter, Total harmonics distortion, Electrical and electronic engineering, Energy science and technology

## Abstract

The power factor correction converter is the function of the front-end converter, followed by the DC–DC converter of the electric vehicle charger. It improves the power factor and regulates the output voltage and current. This research article proposes the Golden Eagle optimization for fractional order PI (FOPI) controller for Single Ended Primary Inductor Converter (SEPIC) power factor correction. The Golden eagle optimization is based on its knowledge of hunting tactics at various degrees of spiral trajectories to catch the prey. The FOPI controller has a broad range of controller parameters that provide better control and performance of the converter. The tuning of the parameters of the FOPI controller is optimized in Golden Eagle Optimization, and the Integral Absolute error with Integral Square error is used for the objective function. The optimized parameters of FOPI compare with the conventional PI controller performance. The SEPIC converter is designed and derived from the state space model by state space averaging, and the reduced model is obtained through the moment matching method. This system is tested under MATLAB/SIMULINK, and simulation results show improved settling time, fast dynamic response, reduction of inrush current, less harmonic distortion, and stability.

## Introduction

The Fuel demand, fossil fuel reserves, and the subsequent increase in fuel prices and emissions of greenhouse gases have put the transportation industry at the epicentre of the climate disaster^1^. Hybrids, electric automobiles, fuel cell vehicles, and solar-powered vehicles contribute to a cleaner environment and less greenhouse gas emissions^2^. Battery chargers are classified as On-board and OFF-board chargers. Off-board charges can only be found at certain places, like fuel stations, and it is possible to charge rapidly with these chargers, but it renders the battery overheated^3^, and the life span is minimal. Concerns about the electric vehicle range and the battery performance are two main obstacles to its broad use. The lack of charging stations and infrastructure also demands On-board chargers (OBC). The PFC converter is an integral part of the OBC charger that transforms the grid supply alternating current into direct current for vehicle battery charging. The boost converter has attracted widespread attention, and the issues with bulky components require a filter at the output and high voltage stress^4^. The Buck converters are less expensive and more efficient, but dead angle issues mainly influence them in supply voltage and need an input filter^5,6^. DC–DC Cuk converters are good at isolation, current limiting, and short-circuit protection. Moreover, two major drawbacks are the severe current stress felt across the switch and the reversed polarity when loaded^7^. An isolated converter, such as flyback, push–pull, and forward converter, is in AC–DC conversion and has problems with size, transformer core saturation, non-minimum phase and current harmonics drawbacks^8^. Non-isolated converter topologies are often selected because of the absence of transformers, and their benefits include low cost, compactness, lightweight, good efficiency, and minimum losses. The SEPIC converter may alleviate the shortcomings of the Cuk converter, Buck converter, and Boost converter, and it has a faster transient response than other converters. These critical points of the SEPIC converter are more appropriate for power factor correction applications when charging a battery.

The behaviour of DC–DC converters is not linear, and this behaviour changes when the parameters are changed^9^. The small-signal model-based controllers for DC–DC converters are inadequate in meeting the stability requirements, needed regulation, and transient responsiveness for significant load variations. To regulate the output voltage, the cascade control technique for single-stage active power factor correction (PFC) utilizes the use of an outer voltage controller. An inner current controller is also used to shape the input current, and the PI controller is employed for this purpose. Many fine-tuning methods have been proposed to improve the feedback system and be more reliable. The PI and PID controls are well-known and used in many fields. They are easy to set up and work on average^10^. PID controllers are mostly insignificant owing to parametric unpredictability and various disturbances^11^. The fractional order controller is sensitive to both dynamic load and requirements, and it has two extra factors on top of the current PID that make it more flexible while still sturdy. It ensures the optimal result for higher-order non-linear systems without a minimum phase. The capacity of fractional order (FO) controllers to endure unpredictability and load disturbance is an essential feature^12^. Because of their increased adaptability, these controllers can better satisfy design criteria such as phase margin and gain, resistance to parameter variations, and more. Because of these characteristics, fractional order controllers can successfully regulate power converters and other non-linear facilities^13^. In the boost converter^14^, Compared to the integer-order controller, the fractional-order controller performs better in overshoot and recovery time in both simulations and experiments. FOPID controller is implemented to minimize vibration, noise, and harmonic current of three-phase induction motors^15^ and controller parameter design with motor specifications. The fractional order PID (FOPID) control scheme needs more accurate and effective tuning than general tuning methods, and this technique improves settling time, lowers overshooting, and lowers the steady-state error of the system. Many research studies are actively using optimization-based metaheuristic algorithms for power electronic applications.

From the literature survey, the converging time of genetic algorithms (GAs) is higher than average because of the randomness of the cross-over and mutation processes^16^. Particle Swarm Optimization (PSO) is used in PI tuning, but it has a significantly low convergence rate and settling times for the converter^17^. Grey wolf optimization has many constraints over convergence and poor searching performance^9^. The Firefly and bacterial foraging optimization are used for PID controller for the converter side, but it has the drawback of being stuck in several local optima solutions^18^. The Golden Eagle Optimisation Algorithm (GEOA) has benefits over other methods^19^ and is a powerful and bio-inspired optimization technique that can find the best global parameters for the outer and inner controllers of the PFC SEPIC converter. The controllers regulate the output voltage quickly and effectively, and the input current is shaped to improve the power factor at the supply side. Hence, the system performance is enhanced, such as virtually UPF, low percentage of THD, and shorter settling time for line, load, and setpoint variations. The GEO tunned FOPI is used in the closed loop cascade control strategy. The THD level is significantly low value, the power factor reaches near unity, and the initial inrush current at switches and the input side is minimal.

The significant contributions of this work are as follows

- Fast dynamic response on variation in input and load
- Reduction of initial inrush current in switching devices and inductor
- Achieve the high-power factor and lower THD

The GEO fractional-order PI controller design for a PFC SEPIC converter in EV charging introduces a pioneering approach to power electronics and EV charging systems. By integrating the GEO technique with FOPI control, the proposed method enhances the performance and efficiency of power factor correction (PFC) SEPIC converters utilized in EV charging infrastructure. This innovation offers superior adaptability to varying load conditions, increased stability, and optimized energy conversion, thereby advancing the efficacy and sustainability of EV charging technologies. This paper is organized into five parts as follows, “Mathematical modelling and design” discusses converter mathematical modelling and design, "Closed loop operation of PFC converter" discusses the closed loop operation of PFC converter, "Simulation results and discussion" is the simulations results and discussion, and finally, the conclusion about the research studies of this paper in the "Conclusion".

## Mathematical modelling and design

### Modelling of SEPIC converter

**Figure 1:**
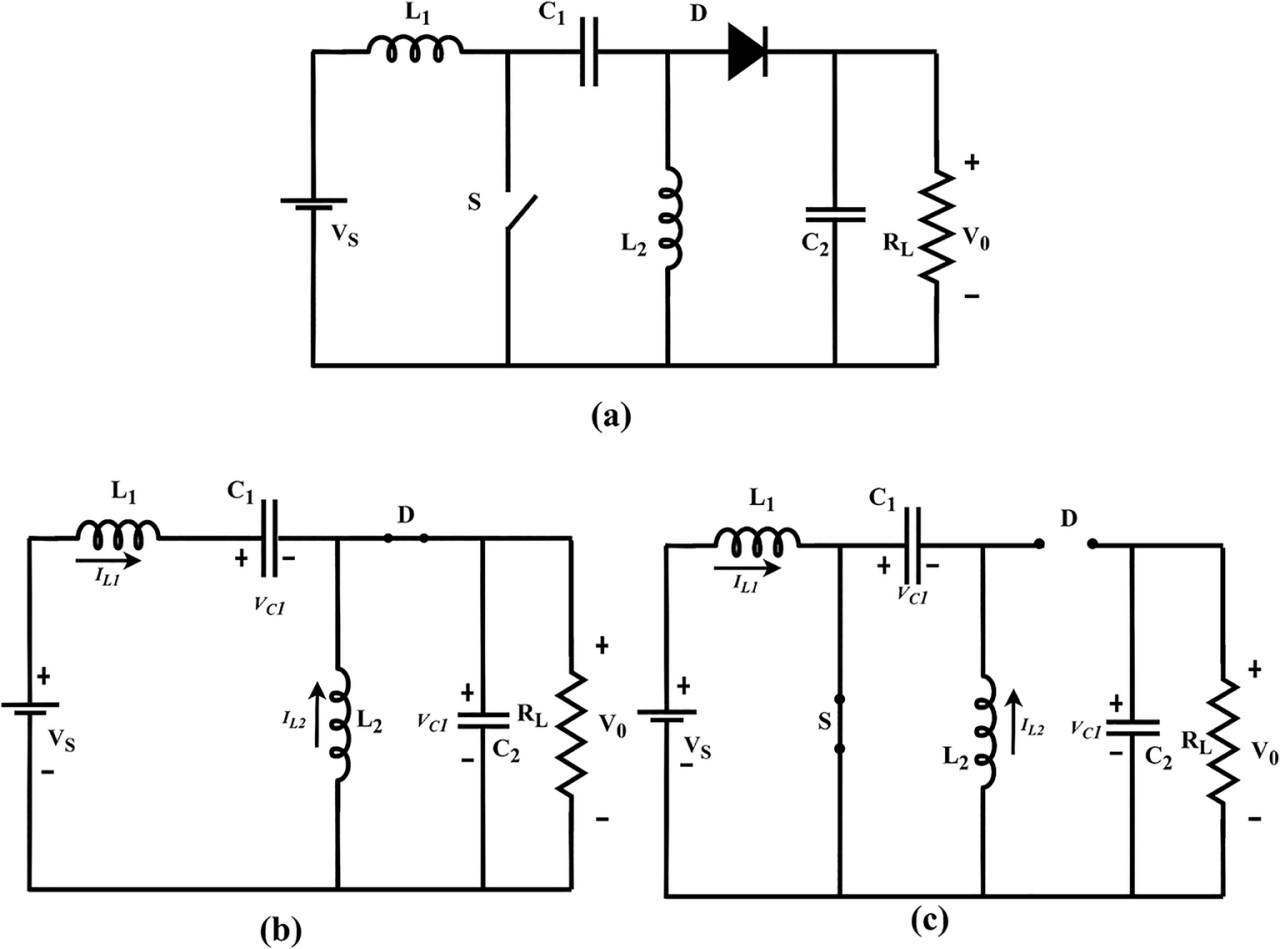
**(a)** SEPIC converter**, (b)** OFF-state**, (c)** ON-state.

The circuit of the SEPIC converter shown in Fig. 1 consists of a MOSFET switch S, diode D, an input and output inductor L_1_ & L_2_, an energy transfer capacitor C_1_, an output filter capacitor C_2_, D is the freewheeling diode and a load resistor (R_L_). When the switch S closes, i_L1_ flows through the inductor L_1_ and charges. Equations (1), (2), and (3) represent the steady state and transient currents and voltages. The state model of the SEPIC converter is derived to obtain the mathematical model of the converter by considering that the switch is ‘ON’, (i.e.) S = 1 during the MOSFET switch conduction subinterval and switch is ‘OFF’ (i.e.) S = 0 during the diode conduction subinterval. State-space averaging is used to depict the dynamics of the converter, and the state equations at switch S = 1 and S = 0 are combined using as follows:

1 $$\left[\begin{array}{c}\frac{d{I}_{L1}}{dt}\\ \frac{d{I}_{L2}}{dt}\\ \frac{d{V}_{C1}}{dt}\\ \frac{d{V}_{C2}}{dt}\end{array}\right]=\left[\begin{array}{cccc}0& 0& 0& 0\\ 0& 0& \frac{1}{{L}_{2}}& 0\\ 0& \frac{-1}{{C}_{1}}& 0& 0\\ 0& 0& 0& \frac{-1}{{R}_{L}{C}_{2}}\end{array}\right]\left[\begin{array}{c}{I}_{L1}\\ {I}_{L2}\\ {V}_{C1}\\ {V}_{C2}\end{array}\right]+\left[\begin{array}{c}\frac{1}{{L}_{1}}\\ 0\\ 0\\ 0\end{array}\right]\left[{V}_{in}\right]$$

2 $$\left[\begin{array}{c}\frac{d{I}_{L1}}{dt}\\ \frac{d{I}_{L2}}{dt}\\ \frac{d{V}_{C1}}{dt}\\ \frac{d{V}_{C2}}{dt}\end{array}\right]=\left[\begin{array}{cccc}0& 0& \frac{-1}{{L}_{1}}& \frac{-1}{{L}_{1}}\\ 0& 0& 0& \frac{-1}{{L}_{2}}\\ \frac{1}{{C}_{1}}& 0& 0& 0\\ \frac{1}{{C}_{2}}& \frac{1}{{C}_{2}}& 0& \frac{-1}{{R}_{L}{C}_{2}}\end{array}\right]\left[\begin{array}{c}{I}_{L1}\\ {I}_{L2}\\ {V}_{C1}\\ {V}_{C2}\end{array}\right]+\left[\begin{array}{c}\frac{1}{{L}_{1}}\\ 0\\ 0\\ 0\end{array}\right]\left[{V}_{in}\right]$$

3 $${V}_{0}=[0 0 0 1]\left[\begin{array}{c}{I}_{L1}\\ {I}_{L2}\\ {V}_{C1}\\ {V}_{C2}\end{array}\right]$$

4 $$A=\left[\begin{array}{cccc}0& 0& \frac{-(1-d)}{{L}_{1}}& \frac{-(1-d)}{{L}_{1}}\\ 0& 0& \frac{d}{{L}_{2}}& \frac{-(1-d)}{{L}_{2}}\\ \frac{(1-d)}{{C}_{1}}& \frac{-d}{{C}_{1}}& 0& 0\\ \frac{(1-d)}{{C}_{2}}& \frac{(1-d)}{{C}_{2}}& 0& \frac{-1}{{R}_{L}{C}_{2}}\end{array}\right]; B=\left[\begin{array}{c}\frac{1}{{L}_{1}}\\ 0\\ 0\\ 0\end{array}\right];C=\left[\begin{array}{cccc}0& 0& 0& 1\end{array}\right]; D=\left[\begin{array}{cccc}0& 0& 0& 0\end{array}\right]$$

5 $$\dot{X}=AX+BU$$

6 $$Y=CX+DU$$

7 $$\frac{{V}_{0}}{{V}_{in}}=\frac{d}{1-d}$$

8 $$\frac{{I}_{in}}{{I}_{0}}=\frac{d}{1-d}$$

9 $$T.F={C(sI-A)}^{-1}[\left({A}_{1}-{A}_{2}\right)*X+\left({B}_{1}-{B}_{2}\right)*{V}_{in}]$$

The duty cycle of the ON and OFF state of the switch is indicated as d and (d-1), and by replacing the derived parameter values in the equivalent state-space matrices, which are subsequently substituted in the formula that follows, the fourth-order transfer function of control to the output of the proposed DC-DC SEPIC is obtained.

### Design of SEPIC converter

The following section explains the design of the SEPIC converter and the components values are listed in Table 1. The input supply (Vin) is 220 V AC, while the output voltage (V_o_) is 60 V, and the high-frequency input filter capacitor and inductor design^20^ is

**Table 1:** Design parameter.

Parameter	Value
Input voltage	220 Vrms
Output voltage	60 V
Duty ratio	0.202
Inductor L1/L2	2 mH
Capacitor C1/C2	10 µF/8000 µF
Switching frequency	30 kHz
Input filter capacitor C_f_	10 µF
Input filter inductor L_f_	2.5 mH
Output power	460 W

10a $${C}_{f}=\frac{{I}_{p}tan\theta }{2\pi *f*\Delta *{V}_{p}}$$

10b $${L}_{f}=\frac{1}{4{\pi }^{2}*{{f}_{c}}^{2}*{C}_{d}}$$

Vp and Ip are peak voltage and current of input supply and for high power factor θ value is chosen for 1. ${f}_{c}$ is the cutoff frequency chosen between grid frequency 50 Hz and switching frequency 30 kHz.

The Duty cycles the converter is calculated from

11 $$D=\frac{{V}_{0}}{{V}_{0}+{V}_{in}}$$

12 $$\text{Ripple current }{\Delta I}_{L}={I}_{out}*\frac{{V}_{out}}{{V}_{in min} }*40\%$$

The inductor L_1_ and L_2_ is calculated from the equation

13 $${L}_{1}={L}_{2}={L}=\frac{D*{V}_{in}}{{\Delta I}_{L1}*{f}_{s}}$$

The coupling capacitor is calculated from

14 $${C}_{1}\ge \frac{{I}_{L2}*D}{{f}_{s}*{V}_{C}}$$

The output capacitor is calculated from

15 $${C}_{2}\ge \frac{{P}_{o}}{4*\pi *{f}_{s}*{V}_{o}{*\Delta V}_{o}}$$

16a $${G}_{vd}=\frac{{v}_{o}}{d}=\frac{-60{S}^{3}+1.485*{10}^{6}{S}^{2}-6.06{10}^{8}S+3.715*{10}^{13} }{0.05513{S}^{4}+0.798{S}^{3}+ 1.872*{10}^{6}{S}^{2}+2.704*{10}^{7}S+9.973*{10}^{10}}$$

16b $${G}_{id}=\frac{{i}_{L}}{d}=\frac{ 60 {s}^{3}+ 1.554*{10}^{5} {s}^{2}+ 6.082*{10}^{8} s + 1.754*{10}^{10} }{0.000404{s}^{4}+ 0.005848{s}^{3}+ 1.372* {10}^{4}{s}^{2}+ 1.981*{10}^{5}s +{7.309*10}^{8}}$$

Examining and creating a large-scale model are challenging tasks that lead to ongoing efforts to simplify the higher-order model and prevent realistic computing efforts to provide appropriate results from traditional research, modelling, control, design, and computation strategies^21^. For developing controls, a lower-order model is better than a higher-order model^22^, and to implement the controller, the above fourth-order equation is reduced to the second-order system by the moment matching method to reduce computational complexity and preserve the original properties. For reduction techniques, the power series expansion is obtained using the long division method from Eq. (16a) as follows:

17 $${G}_{vd(q)}=372.5-0.101s-6.953*{10}^{-3}{s}^{2}+3.78*{10}^{-6}{s}^{3}+1.295*{{10}^{-7}s}^{4}+\dots$$

18 $$G\left(s\right)={C}_{0}+{C}_{1}s+{C}_{2}{s}^{1}+{C}_{3}{s}^{2}+{C}_{4}{s}^{3}+\dots \dots \dots +\dots$$

The power series expansion coefficients are the C_0_, C_1_, C_2_, C_3_ and C_4_, and the second-order system structure is as follows.

19 $$r\left(s\right)=\frac{{a}_{21}+{a}_{22}S}{1+{a}_{12}S+{a}_{13}{S}^{2}}$$

20 $${\widehat{a}}_{1}={\left[\begin{array}{cc}-{c}_{1}& -{c}_{0}\\ -{c}_{2}& -{c}_{1}\end{array}\right]}^{-1}*\left[\begin{array}{c}{c}_{2}\\ {c}_{3}\end{array}\right]=\left[\begin{array}{c}{a}_{12}\\ {a}_{13}\end{array}\right]$$

$${a}_{12}=2.7144{*10}^{-4},{a}_{12}=1.8739*{10}^{-5}$$

21 $${\widehat{a}}_{2}=\left[\begin{array}{c}{c}_{0}\\ {c}_{1}\end{array}\right]-\left[\begin{array}{cc}0& 0\\ {c}_{0}& 0\end{array}\right]*\left[\begin{array}{c}{a}_{12}\\ {a}_{13}\end{array}\right]=\left[\begin{array}{c}{a}_{21}\\ {a}_{22}\end{array}\right]$$

$${a}_{21}=372.5,{a}_{22}=-0.2021$$

By substituting the respective values in the Eq. (20) and (21), the reduced order transfer function is

22 $${G}_{vdr}=\frac{-0.2021 s + 372.5}{1.8739*{10}^{-5} {S}^{2} + 2.7144*{10}^{-4}s + 1}$$

**Figure 2:**
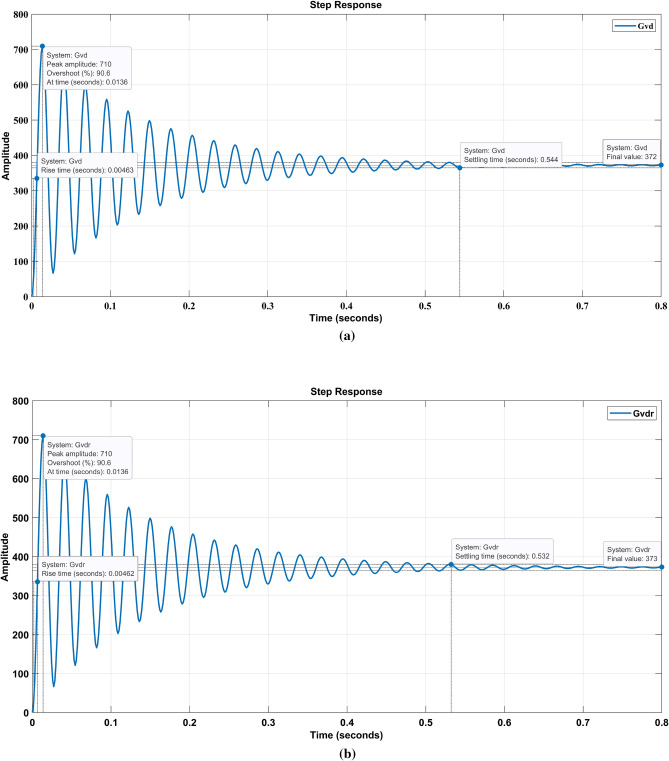
**(a)** Step response of 4th order and (**b)** reduced order SEPIC.

Figure 2a, b shows the step response of fourth order and the reduced order transfer function of the SEPIC converter. The peak overshoot M_P_ is 90.6%, settling time T_S_ is 0.532 s, and rise time T_R_ is 0.00462 s compared to the actual fourth order system step response, which is identical to the reduced-order system.

## Closed loop operation of PFC converter

### Closed loop operation of converter

**Figure 3:**
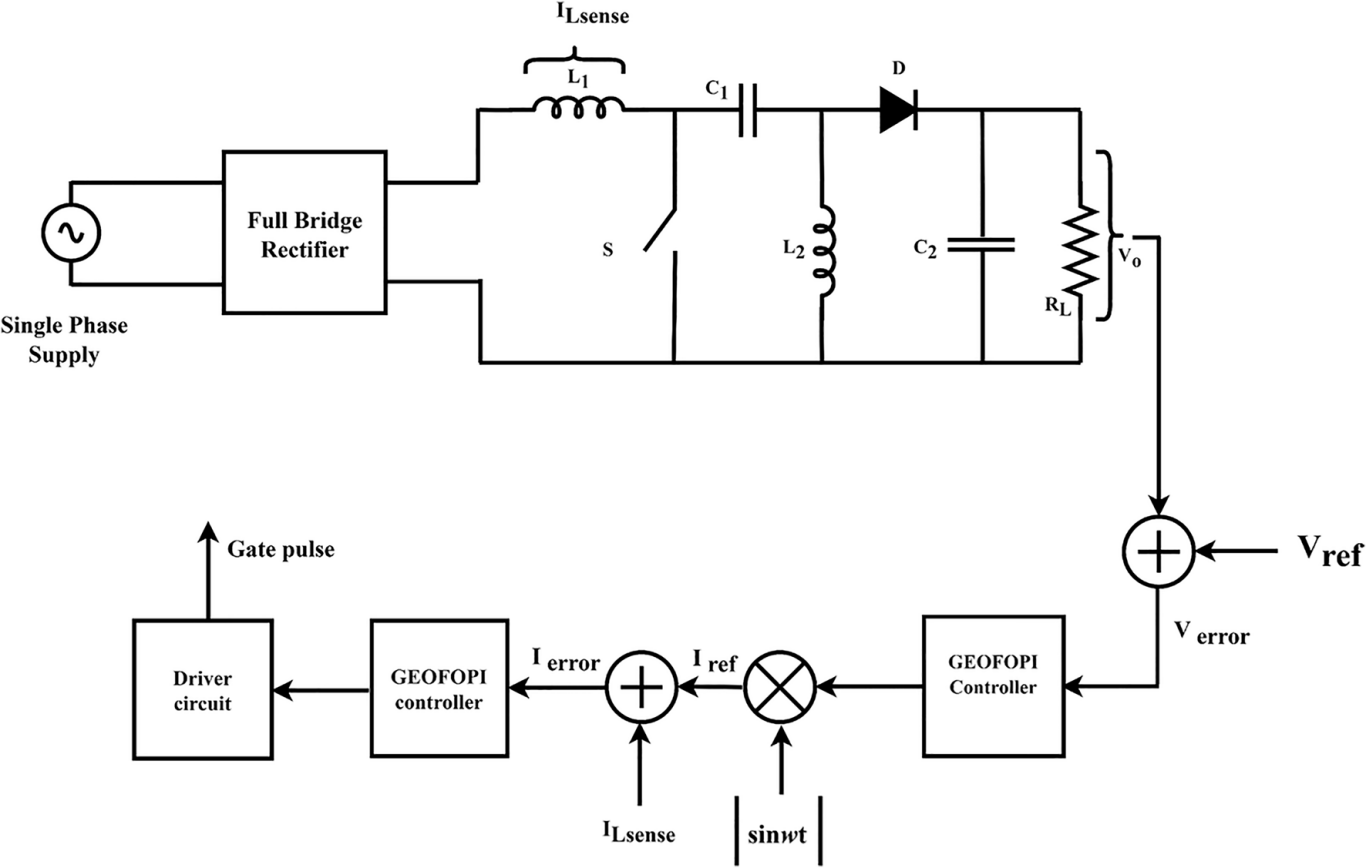
Block diagram of PFC controller.

The closed-loop control of the proposed system uses a cascade control strategy in Fig. 3. The inner and outer loop is optimized fractional proportional integral control to improve the power factor at AC supply while voltage control gives reference voltage. The input single-phase AC supply is converted into a pulsating DC supply through a diode bridge rectifier, which causes a low power factor, less efficiency, and high harmonic distortion. The SEPIC converter is used for power factor correction, and the output voltage (V_o_) is sensed and compared with the reference voltage (V_ref_). The error voltage is an input to the voltage controller, and the output is multiplied by a pulsated absolute sin wave template. The output values of the voltage controller are the reference current to the current controller, which generates the necessary pulse width modulation pulse for the MOSFET switch to improve the power factor at single phase supply.

### Design of fractional order PI controller

A standard PID controller, that is based on fractional calculus, additionally produces the FO-PID approach, and controllers is upgraded to a controller is called fractional order FOPI and the transfer function of fractional order PI controller (C_S_) equation is Eq. (23)

23 $${C}_{S}=({K}_{P}+\frac{{K}_{I}}{{s}^{\lambda }})$$

**Figure 4:**
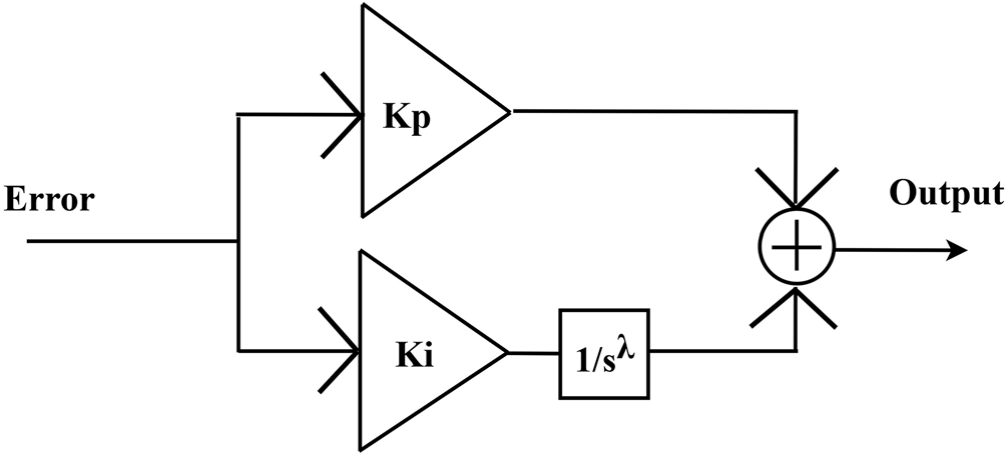
FOPI structure.

For the FOPI, three parameters, K_P_, K_I,_ and λ, are the tuning parameters of the controller. The FOPI controller improves the system stability, robustness and effectiveness by adding the fractional integral λ. The additional tuning parameter (λ) gives the degrees of freedom for tuning the controller action more accurately than the PI controllers, which is represented in the Fig. 4.

### Golden eagle optimization of controllers

In General, the optimizations are used to fine-tune the controller parameters to improve the performance. A new bio-inspired method called Golden Eagle Optimization (GEO) created the voltage controller. GEO showed faster convergence times than earlier optimization techniques in numerous applications. This algorithm's inspiration comes from the golden eagle^19^, whose intelligence was used to model its turning speed at various points along its circular path while hunting. It has been observed that golden eagles are more likely to cruise than attack during the early phases of a hunt, while the opposite is valid during the latter stages of the search. GEO is based on the spiral movement of the Golden Eagle, where each eagle remembers the best places, it has visited. The eagle attack is shown by a path from where it is now to where it wants to catch its food, and several steps before catching the target foods are as follows.

#### Attack

24 $${\overrightarrow{A}}_{i}={\overrightarrow{X}}_{f}^{*}-{\overrightarrow{X}}_{i}$$

${A}_{i}=[{a}_{1},{a}_{2},\dots \dots ,{a}_{3}]$, ${A}_{i}$-the attack vectors,${X}_{i}=[{x}_{1},{x}_{2},\dots \dots ,{x}_{m}]$, ${X}_{i}$- the decision variable vector

${\overrightarrow{X}}_{f}^{*}=[{x}_{1}^{*},{x}_{2}^{*},\dots \dots ,{x}_{m}^{*}]$,${X}_{f}^{*}$-is best location of travelled by the eagle so far. The attack vector navigates the population of the eagles towards the best locations.

25 $$d=\sum_{j=1}^{n}{h}_{j}{x}_{j}$$

### Searching (cruise)

The search vectors is determined through

$\overrightarrow{H}=[{h}_{1},{h}_{2},{h}_{3},\dots ..,{h}_{n}]$ is the normal vector,$X=\left[{x}_{1},{x}_{2},{x}_{2 },\dots .,{x}_{n}\right]$, is the variable vector

26 $$\sum_{j=1}^{n}{a}_{j}{x}_{j}=\sum_{j=1}^{n}{a}_{j}^{t}{x}_{j}^{*}$$

${a}_{j}$ is the element of ${j}$ th attack vector, $t$—is an iteration value

27 $${\overrightarrow{C}}_{i}=({c}_{1},{c}_{2},{c}_{3},\dots \dots ..,{c}_{k},\dots ..,{c}_{n})$$

Cruise vector ${C}_{k}$ is perpendicular to the attack vector, and it is tangent to the circular path of the eagle,$k$ represents the fixed variable index, ${a}_{k}$ is the element of ${k}$ th attack vector.

28 $${C}_{k}=\frac{d-\sum_{j,j\ne k}{a}_{j}}{{a}_{k}}$$

The eagle's displacement is built up of its attack and cruise vectors, and its updated location is calculated by Eq. (29)

29 $${x}^{t+1}={x}^{t}+\Delta {x}_{i}^{t}$$

#### Change the location

30 $$\Delta {x}_{i}={\overrightarrow{r}}_{1}{p}_{a}\frac{{\overrightarrow{A}}_{i}}{|| {\overrightarrow{A}}_{i}||}+{\overrightarrow{r}}_{c}{p}_{c}\frac{{\overrightarrow{C}}_{i}}{|| {\overrightarrow{C}}_{i}||}$$

**Figure 5:**
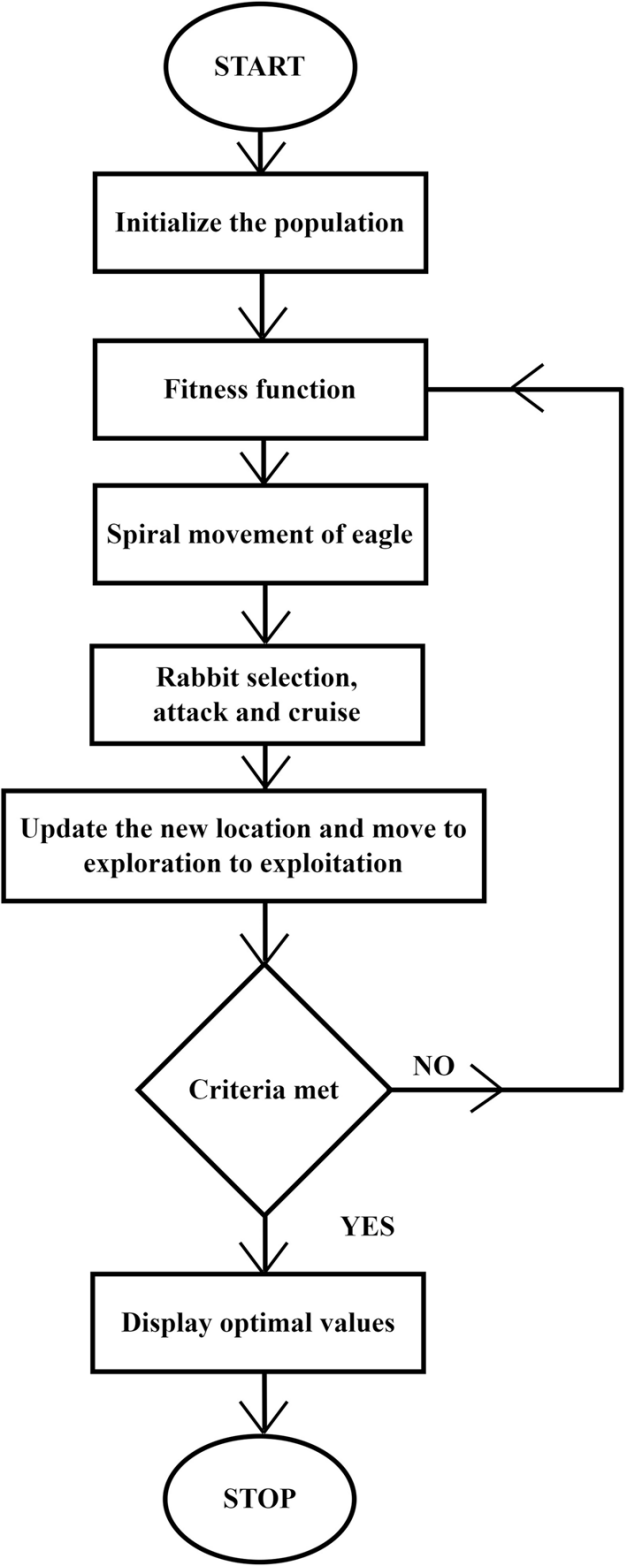
Golden eagle optimization flowchart.


$||{\overrightarrow{A}}_{i}||=\sqrt{\sum_{j=1}^{n}{a}_{j}^{2}}$, $|| {\overrightarrow{C}}_{j}||=\sqrt{\sum_{j=1}^{n}{C}_{j}^{2}}$—are the Euclidean norms of attack and cruise vector

31 $${p}_{c}={p}_{c}^{0}-\frac{t}{T}|{p}_{c}^{T}-{p}_{c}^{0}|$$

32 $${p}_{a}={p}_{a}^{0}-\frac{t}{T}|{p}_{a}^{T}-{p}_{a}^{0}|$$

t—represents the current iteration, T—represents the iteration range, ${p}_{c}^{0}$ and ${p}_{a}^{T}$ represents the initial and final values for propensity to reach ${p}_{a}^{T}$, ${p}_{c}^{0}$ and ${p}_{c}^{T}$ represents affinity to descent of the prey ${p}_{c}$. This optimization works on ${p}_{a}$ to ${p}_{c}$ changing from search to exploitation. The algorithm default starts with a high *p*_*c*_ and a low *p*_*a*_. As the repetitions continue, *p*_*a*_ is raised while *p*_*c*_ is lowered. The proposed optimization flow chart describes the optimization techniques of the eagle, as shown in Fig. 5.

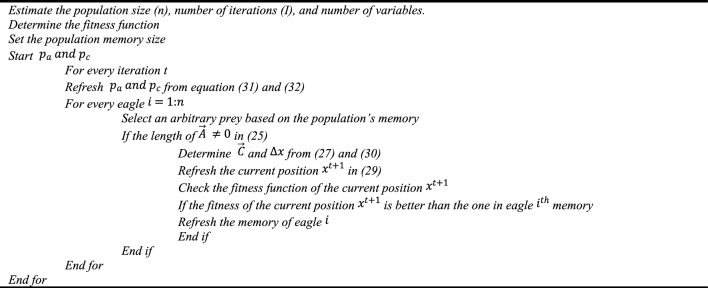


### Optimization of voltage controller

**Figure 6:**
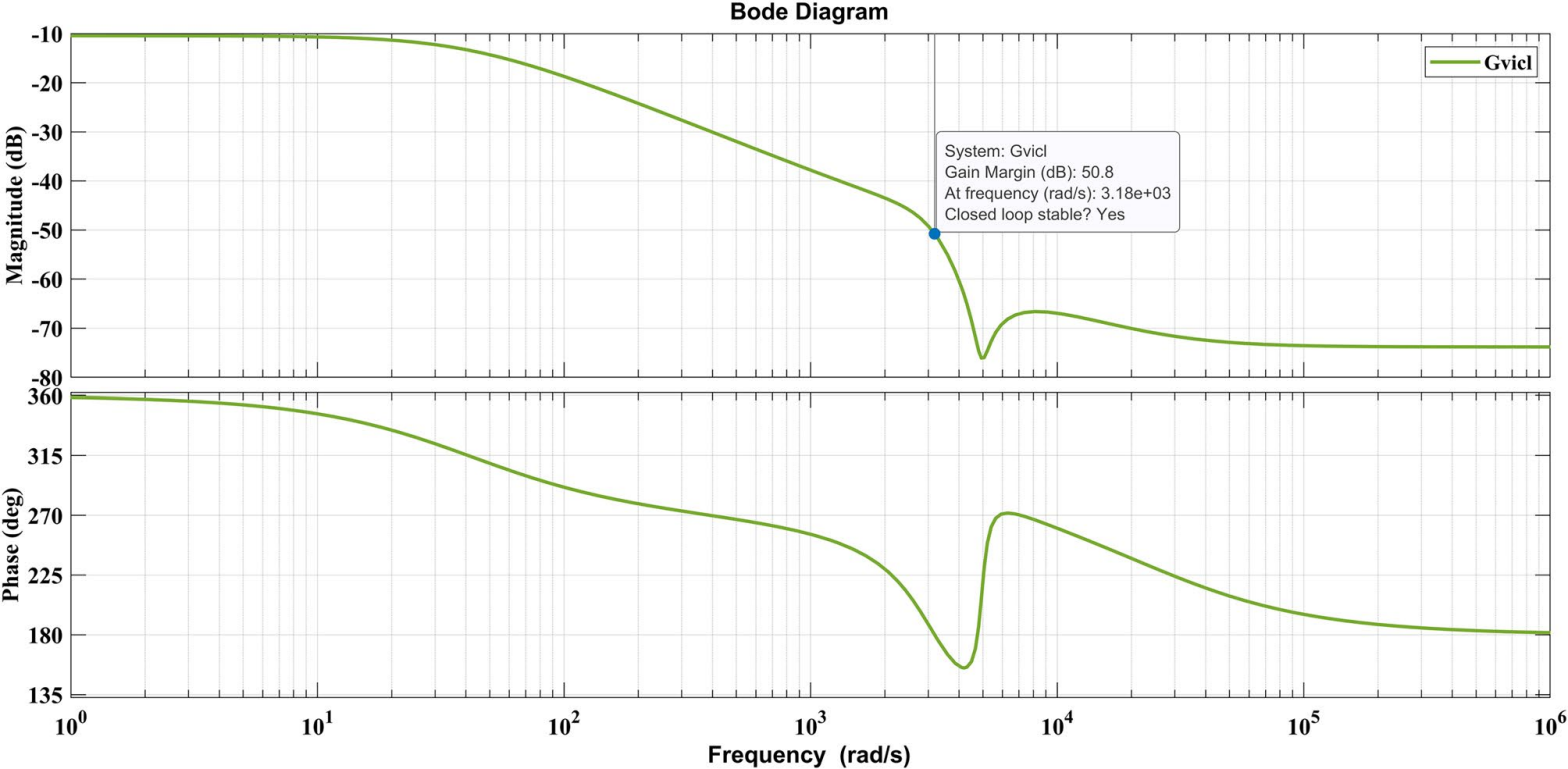
Frequency response of voltage controller.

The conventional method of Ziegler- Nicholas (Z-N) method is used to find the K_P_ and K_I_ values for optimization constraints and ultimate period $\text{Pcr}=7*{10}^{-3}\text{ s}$ and critical gain value $\text{Kcr}=0.0389$ obtained. By substituting these values to determine the PI controller values from the Table 2, the propositional and integral gain values are ${K}_{P}$=0.000154 and ${K}_{I}$ = 0.12. In the cascade control technique, the voltage controller should work much slower than the inner current loop, and the bandwidth is one-tenth of the inner loop current controller bandwidth^23,24^. The optimization range for proportional and integral gain values is selected using Z-N approach. The design of FOPI is based on fixing the proportional value constant while other parameters vary^25^. The optimization technique calculates tuning parameters based on the system stability and ensures a long settling time. The long settling time causes a delaying steady state, and the quick settling time causes the system to become unstable, which causes a high current that damages the system. The tuning parameter ranges are chosen as in Table 3, and suitable PI parameters are determined through the golden eagle optimization algorithm. Table 4 shows the optimized parameters of FOPI values and their impact on total harmonic distortion. The optimized parameters obtained from GEO is K_P_ = 1.04812*10^–5^, K_I_ = 0.01788, and lambda (λ) value is 1.01. The respective closed-loop response is stable with gain margin (gm) = 50.8, and frequency domain of bode plot as shown in Fig. 6 for the outer loop transfer function of output voltage to inductor current is Eq. (33)

**Table 2:** Ziegler Nicholas tuning.

Controller	Kp	Ti	Td
P	0.5Kcr	–	–
PI	0.45Kcr	Pcr/1.2	–
PID	0.6Kcr	0.5Pcr	0.125Pcr

**Table 3:** Optimization parameters of voltage controller.

Parameter	Value
No of search agents	50
No of iterations	100
Range of K_P_ values	0–0.009
Range of K_I_ values	0–0.09
Range of lambda (λ) values	0.5–2

**Table 4:** Optimized FOPI parameters and performance.

Controlling method	K_P_	K_I_	λ	THD %
Z-N PI	0.000154	0.12	–	3.07
GA-FOPI	0.00013607	0.274	1.11	2.81
PSO-FOPI	0.000012192	0.158	1.1	2.64
HBO-FOPI	0.000025	0.0213	0.83	2.43
GEO-FOPI	0.0000104812	0.01788	0.995	1.85

33 $${G}_{vi}=\frac{-0.000404 {s}^{3}+ 9.998{s}^{2}-4080s +2.499{*10}^{8} }{0.05513{s}^{3}+ 142.8{s}^{2}+ 5.588*{10}^{5}s + 1.612*{10}^{7}}$$

### Optimization of current controller

**Figure 7:**
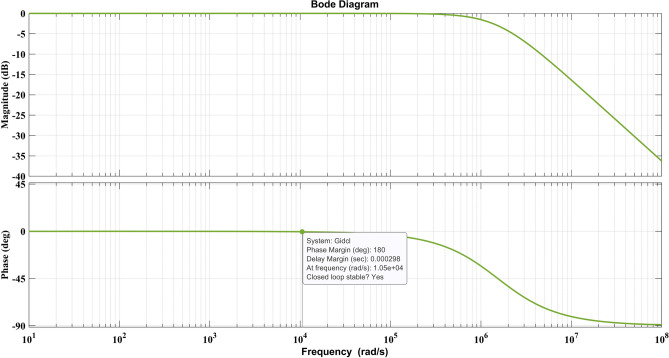
Frequency response of current controller.

For the current controller design, the transfer function of inductor current to duty ratio^26^ is obtained (16b) and the moment matching method in the “Design of SEPIC converter”, the reduced order equations are obtained as follows (34). The current controller optimization parameter ranges are in Table 5. The obtained values from optimization are K_P_ = 34.6, K_I_ = 750.89 and lambda (λ) = 1.01. The transfer function improves the power factor and limits the inductor current. The current controller closed loop frequency response is shown in Fig. 7, with stable conditions for obtained controller parameters and current controller is stable condition.

**Table 5:** Optimization parameters of current controller.

Parameter	Range of Value
Population size	50
No of iterations	100
K_P_	0–100
K_I_	0–1000
lambda (λ)	0.9–2

34 $${G}_{idr}=\frac{ 6.082* {10}^{8}s + 1.754*{10}^{10}}{ {1.37*{10}^{4}S}^{2} + 1.978*{10}^{5}s + 7.309*{10}^{8}}$$

## Simulation results and discussion

**Figure 8:**
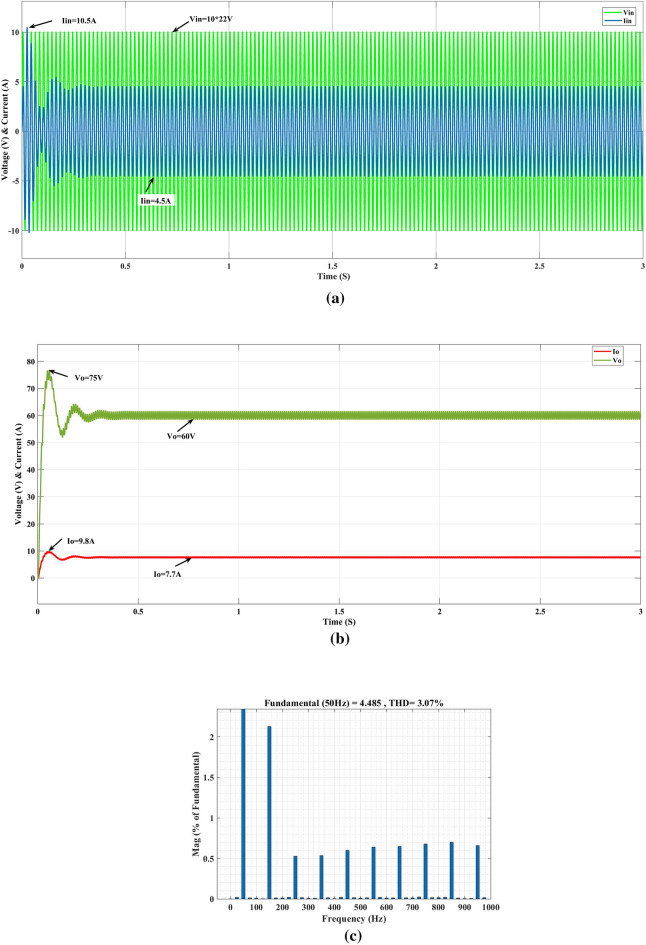
Converter response on PI (**a**) input, (**b**) output, (**c**) THD.

In steady-state simulation, for the input AC supply voltage (V_in_) is 220 V rms, desired output dc voltage (V_o_) is 60 V, and load resistance (R_L_) is 8 Ω, the simulated waveforms of the input and output voltages and currents are shown in Fig. 8. The output voltage and current are settling at 0.2 s with an output current (I_o_) of 7.7A, an output voltage (V_o_) of 60 V, and an input AC supply voltage (V_in_) of 220 V with a current (I_in_) of 4.5A.

### Efficiency calculation of converter

35 $$\text{Conduction loss of MOSFET }({P}_{S})={I}_{rms}^{2}*{R}_{DS on}*{D}_{max}$$

I_rms_ is the rms current of MOSFET switch, R_DSON_ is the body resistance of the switch, D_max_ is the Duty cycle. P_S_ = 4.412 W

36 $$\text{Conduction loss of diode }\left({\text{P}}_{\text{D}}\right)={V}_{VFD}*{I}_{Avg}+{I}_{rms}^{2}*{R}_{d}$$

V_VFD_ is the diode forward voltage drop, I_rms_ is the rms current of the diode, I_Avg_ is the average current value of the diode. R_d_ is the diode dynamic resistance. Pd = 10.21 W

37 $${\text{Inductor loss }}\left( {{\text{P}}_{{\text{L}}} } \right) = {\text{P}}_{{\text{W}}} + {\text{ P}}_{{{\text{Core}}}}$$

$${\text{Wire loss }\left(\text{PW}\right)=R}_{W}*{I}_{L}^{2}$$

I_L_ is the current through the inductor, R_W_ is the resistance of the wire.

$${\text{Core loss }\left(\text{PCore}\right)=K}_{1}{f}^{x}{B}^{y}{V}_{e}$$

P_L_ = 2.106 W

38 $$\text{Capacitor loss }\left(\text{PC}\right)={I}_{C}^{2}*ESR$$

I_C_  is the  current in the capacitor, ESR is the effective series resistance of the capacitor. P_C_ = 0.48 W

39 $${\text{Total conduction loss}} = {\text{P}}_{{{\text{Con}}}} + {\text{ P}}_{{\text{D}}} + {\text{P}}_{{\text{L}}} + {\text{P}}_{{\text{C}}}$$

Total conduction loss T_Total_ = 4.412 + 10.21 + 2.106 + 0.48

40 $$\text{Efficiency of the converter}=\frac{Output power}{Output power+Losses}=96.39$$

### Closed loop response of PFC converter

**Figure 9:**
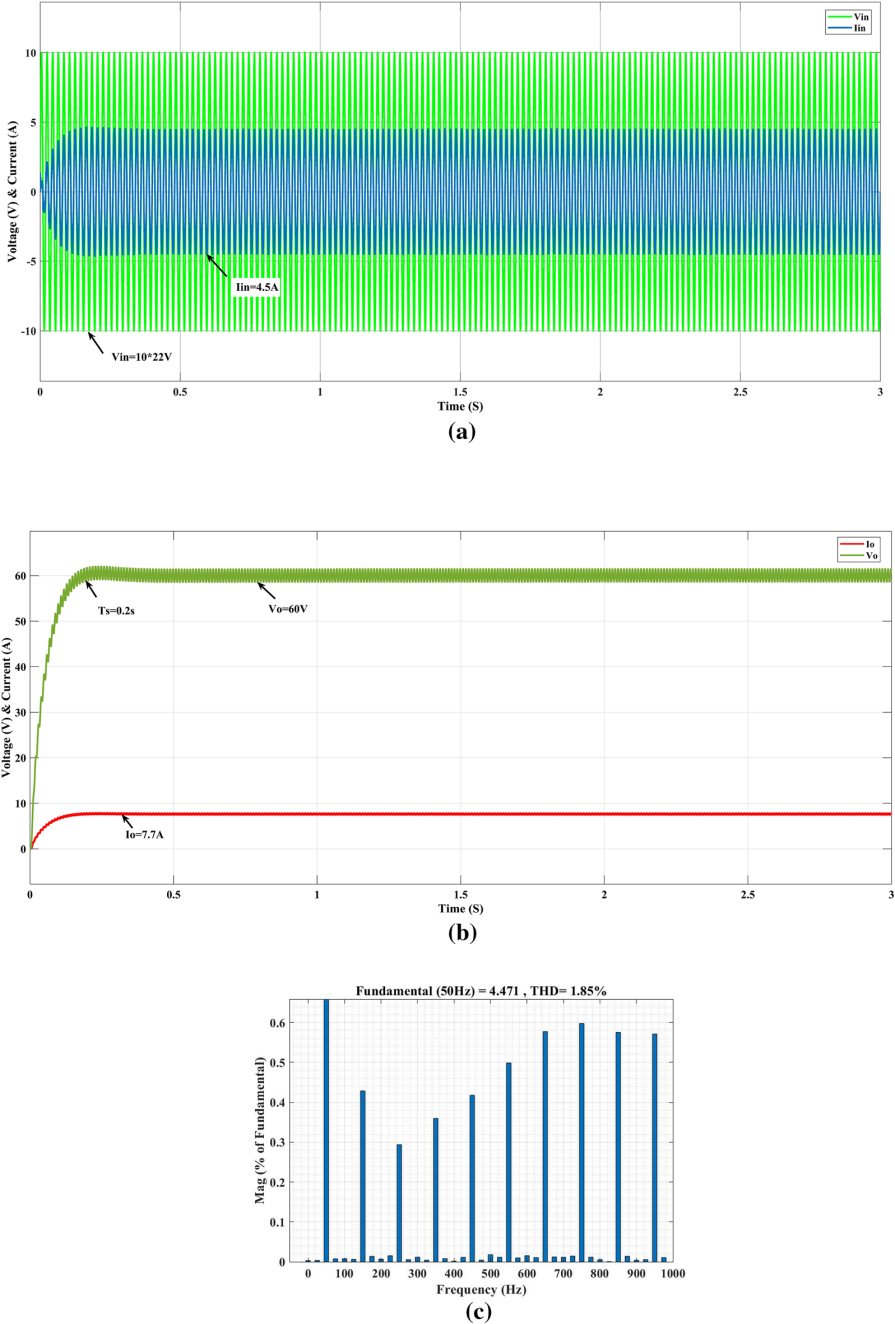
Converter response on GEO-FOPI (**a**) input, (**b**) output and (**c**) THD.

In Fig. 8. shows the closed-loop response of the conventional PI controller. The input supply current (I_in_) is 4.5A, and the voltage (V_in_) is 220 V. During the initial period, the input current (I_in_) is 10.5A at AC supply side and settles in 4.5A in 0.3 s. The THD level is 3.07%, and the output voltage (V_o_) and current (I_o_) reach 75 V with 9.8A initially and settle after 0.3 s with 60 V with 7.8A. Figure 9a shows the GEO-FOPI controller performance of the converter; the AC supply reaches the steady state at time T = 0.2 s and converter output steadily vary and reaches the output voltage (V_o_) 60V and current (Io) 7.7A at time T=0.2s as shown in Fig. 9b, and total harmonics distortion (THD) is 1.85% obtained, as shown in Fig. 9c.

### Variation of load change

**Figure 10:**
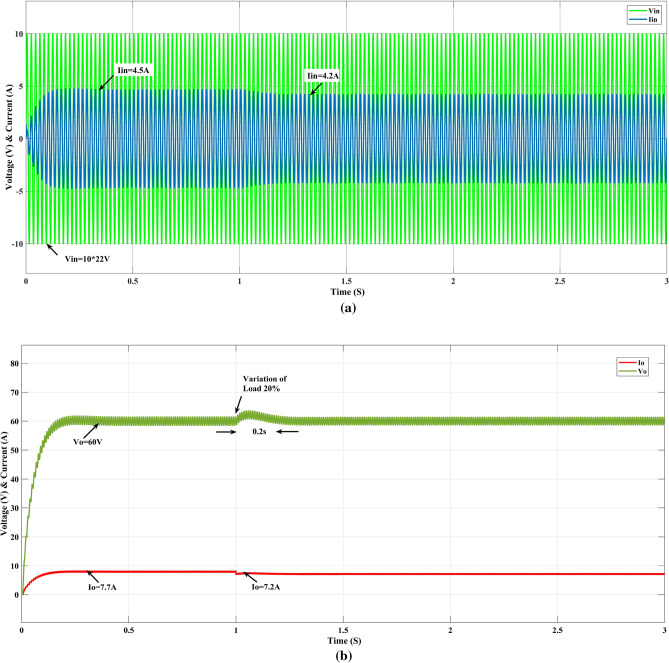
GEO-FOPI response of variation of load (**a**) input and (**b**) output.

Figure 10a shows output load variation at time T = 1 s; the input current (I_in_) is reduced from 4.5 to 4.2 A, the output voltage (V_o_) varies at time T = 1, again settle 60 V at time T = 1.2 s, and output current (I_o_)varies from 7.7 to 7.2 A as shown in Fig. 10b.

### Variation of input voltage

**Figure 11:**
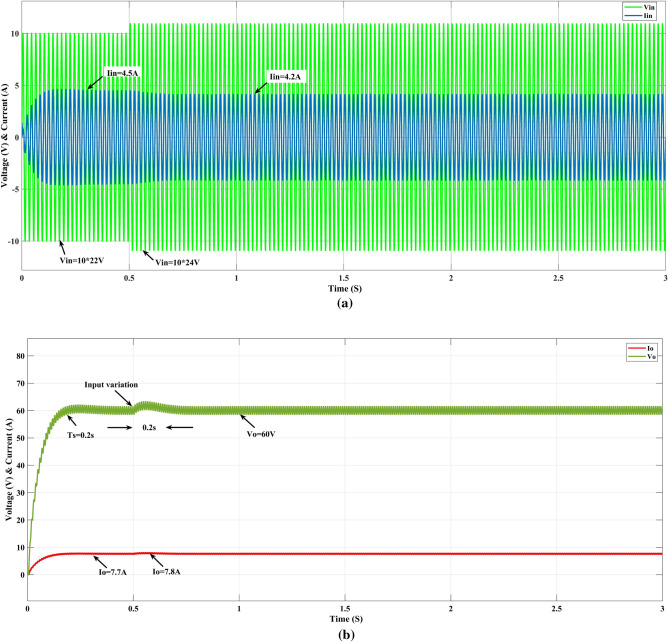
GEO-FOPI response on input variation.

Figure 11 shows the input voltage varied from 220 to 240 V at time T = 1 s, the input current (Iin) reduced from 4.5 to 4.2 A, the output voltage (V_o_) is slightly raised and settled to 60 V after 0.2 s of the time T = 0.5 s, as well as at the same time output current (I_o_) changed from 7.7 to 7.8 A.

### Variation of reference voltage

**Figure 12:**
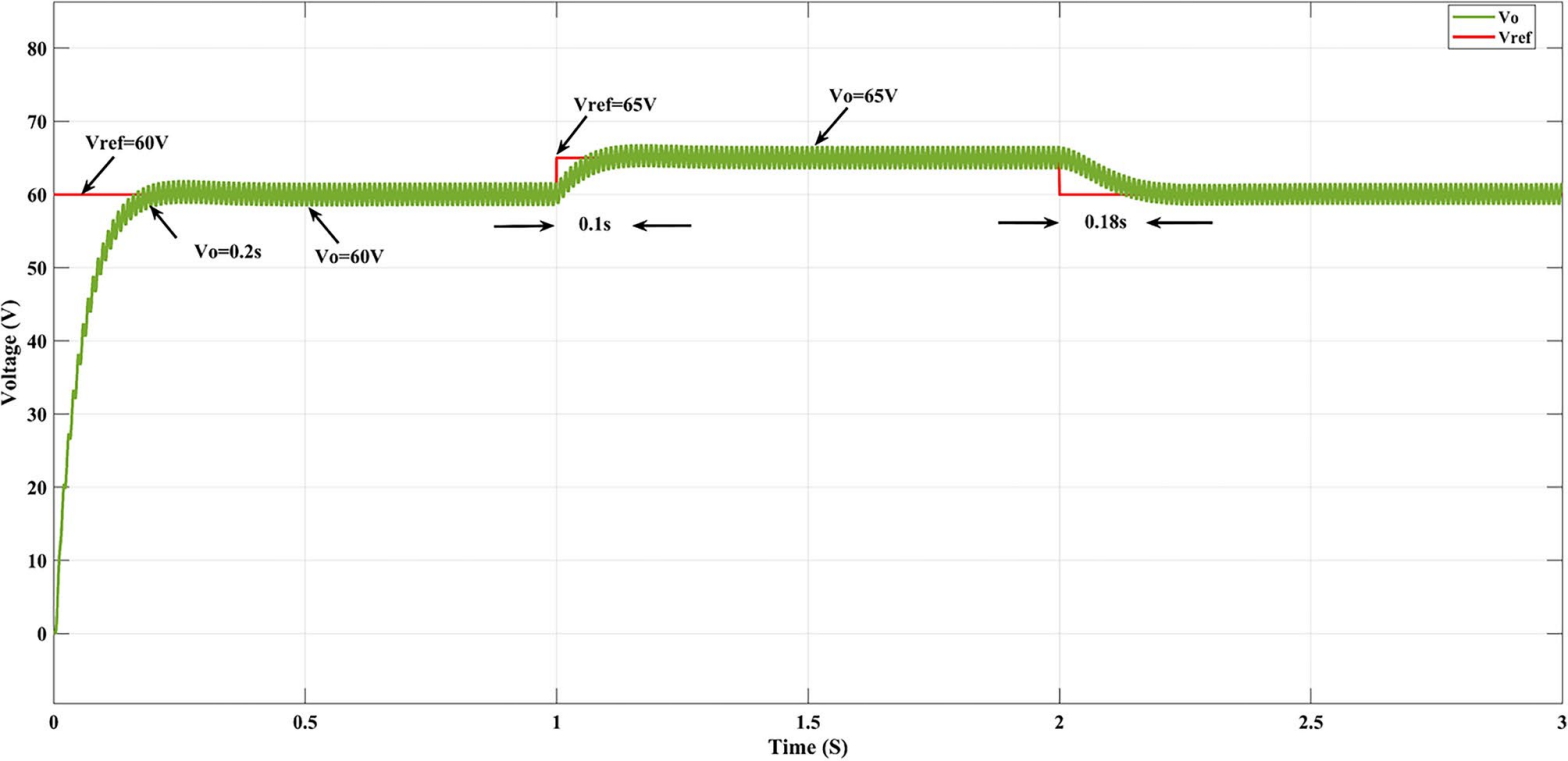
GEO-FOPI controller reference voltage tracking.

Figure 12 shows the output voltage (V_o_) tracking the change reference voltage (V_ref_). When T = 1, the reference voltage (V_ref_) varies from 60 to 65 V, and the output voltage (V_o_) tracks and reaches 65 V at time T = 1.1 s. At time T = 2, the reference voltage (V_ref_) varies from 65 to 60 V, and the converter output voltage reaches 60 V at 2.18 s.

### Current ratings of diode and MOSFET

**Figure 13:**
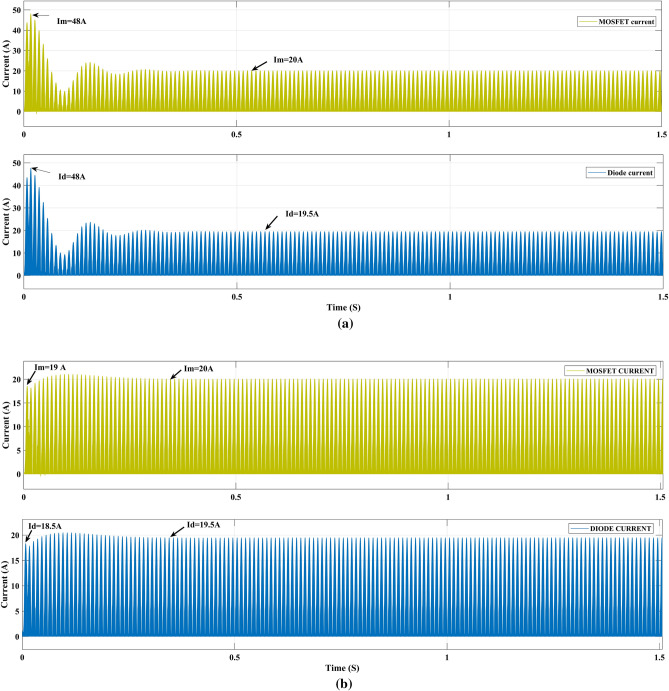
Diode and MOSFET current (**a**) PI, (**b**) GEO-FOPI controller.

The diode and MOSFET current of the PI controller and FOPI controller, Fig. 13a, b initial current reduce significantly from 48 to 18.5 A in the diode as well as from 48 to 19 A reduced in MOSFET. During the initial period, the total conduction loss of both diode and switch loss of 31.642 W is reduced to 18.46 W in the optimized fractional order PI controller. So, the selection of inductor rating is low, and the cost is low compared to the PI controller.

### Current ratings in supply and input inductor

**Figure 14:**
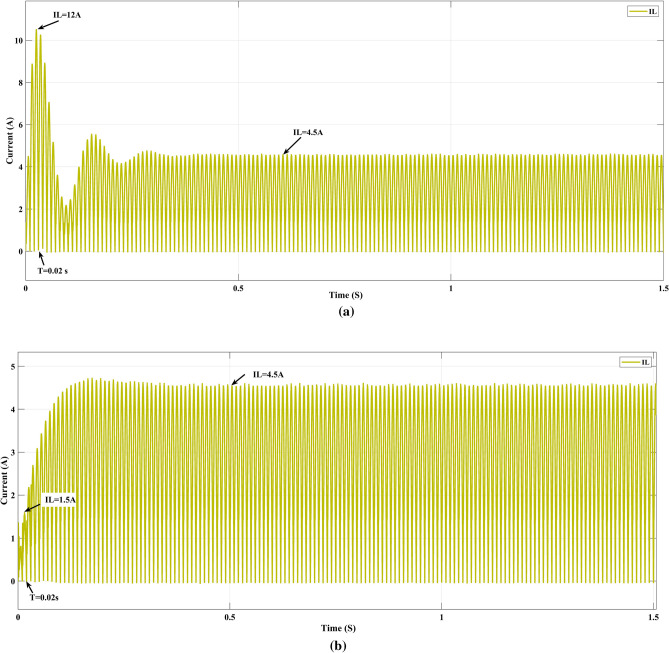
Input inductor current value (**a**) PI, (**b**) GEO-FOPI controller.

Figure 14a, b show the current rating at the inductor (L_1_) is 12 A for PI and 1.5 A for optimized FOPI at time T = 0.02 s, and also the selection of inductor rating and size is low. In Table 6, various controllers with total harmonic distortion are shown, and the Golden Eagle optimization fractional order PI controller gives 1.85%, while other controlling techniques have less dynamic response while fractional order PI controllers provide a fast dynamic response. The golden eagle optimization with fractional order proportional integral controller (GEO-FOPI) offers high harmonic reduction, fast dynamic response, and high-power factor, making it a highly efficient and effective solution compared to other controllers. In the comparison of existing optimization techniques used to evaluate the performance of the PFC SEPIC converter in Table 7, golden eagle optimization gives good results among other optimized methods, and Table 8 comparison of GWO-FOPI and GEO-FOPI shows the GEO-FOPI provides better performance in terms of power factor and efficiency.

**Table 6:** Comparison of proposed controller with existing controlling methods.

Ref	Controller	Converter	Harmonic reduction	Grid detection	Controller design	Power	Dynamic response	Source current THD (%)	Power factor
^27^	SM	Boost	Medium	NA	Complex	500 W	Fast	2.4	0.998
^28^	PI-PI	CUK	Low	NA	Low	–	Slow	2.66	0.99
^29^	SM-PI	SEPIC	Medium	NA	Medium	200 W	Fast	3.169	0.99
^30^	PI	Interleave boost	Low	NA	Medium	1.6 kW	Slow	4.5	–
^31^	PI	Boost	Low	Yes	Low	–	Slow	2.28	–
^7^	PI-LQR	BOCUK	Medium	NO	Medium	200 W	Medium	2.7	0.99
^32^	PSO-PI	Vienna rectifier	Medium	NA	Medium	1.1 kW	–	2.47	0.996
^33^	BC-PI	BOOST	Low	NA	Low	500 W	Medium	4.2	0.994
^34^	PSO-GSA PI-SM	CUK	Low	NO	Complex	1.1 kW	Fast	4.52	0.97
^35^	DE-PI	Boost	Medium	NO	Low	1 kW	Medium	2.23	0.999
^36^	PSO-PI	LUO	Low	NO	Low	–	Medium	8.6	0.97
^37^	PSO-FFPI	Boost	Low	NO	Medium	–	Fast	4.72	0.99
^38^	GWO PID	Bridgeless converter	Low	NO	Low	350 W	Slow	4.45	0.97
GEO method	FOPI	SEPIC	High	NA	Medium	460 W	Fast	1.85	0.9999

**Table 7:** Comparison of PFC SEPIC converter with existing methods.

Controlling method	Gain margin	Settling time	Rise time (s)	Overshoot	Source current THD %	Efficiency	Power factor
Z-N PI	19.6	0.45	0.01	34.459%	3.07	87.67	0.92
GA-FOPI	25.678	0.357	0.025	31.417%	2.81	90.47	0.97
PSO-FOPI	31.3	0.28	0.041	29.070%	2.64	93.01	0.992
HBO-FOPI	42.51	0.25	0.065	13.068%	2.43	93.81	0.995
GEO-FOPI	50.8	0.2	0.09	2.727%	1.85	96.39	0.99998

**Table 8:** Comparison of GWO-FOPI and GEO-FOPI.

Controlling method	K_P_	K_I_	λ	Efficiency	Power factor
^18^ GWO-FOPI	0.08	1.07	1.2	94.5	0.999
GEO-FOPI	0.0000104812	0.01788	0.995	96.39	0.99998

## Conclusion

This article uses the Golden Eagle optimization algorithm for FOPI cascaded controllers for the SEPIC converter's voltage and power factor correction. The reduced order system is obtained from the moment matching method for ease of computational complexity. The simulation of the cascaded loop is executed in MATLAB/Simulink, and the results are examined. The simulation results reveal that the optimized voltage controller and linear quadratic regulator produce a quicker dynamic response with less overshoot than the existing PI Controller. Simulation results shows that optimization of the FOPI controllers makes the system close to the unity power factor (0.9998), initial conduction loss of MOSFET and diode is significantly reduced, and 1.85% of total harmonics distortion is obtained. The FOPI controller significantly reduced the initial inrush current at input from 12 to 1.5 A and switches from 48 to 19 A. The proposed GEO optimized FOPI controller the PFC SEPIC converter reaches the reference output voltage in less settling time for load and input supply disturbance.

## Data Availability

All data underlying the results are available as part of the article, and no additional source data are required.
